# Evaluation of the relationship of treatment and vaccination with prognosis in patients with a diagnosis of COVID-19

**DOI:** 10.1007/s10787-024-01457-4

**Published:** 2024-03-16

**Authors:** Seyma Oncu, Derya Korkmaz

**Affiliations:** 1 Department of Pharmacology, Faculty of Medicine, Afyonkarahisar Health Sciences University, Afyonkarahisar, 03030 Turkey; 2 Department of Infectious Disease, Faculty of Medicine, Afyonkarahisar Health Sciences University, Afyonkarahisar, Turkey

**Keywords:** COVID, Favipiravir, Chloroquine, Vaccine, Prognosis, Elderly

## Abstract

**Purpose:**

Coronavirus disease 2019 (COVID-19) has affected millions of people worldwide and caused mortality. Many factors have been reported to affect the prognosis of COVID-19. In this study, we aimed to investigate the effects of drug therapy and vaccination on prognosis in patients hospitalized with a COVID-19 diagnosis.

**Methods:**

In this single-center, cross-sectional study, data were retrospectively collected from patients receiving inpatient treatment at a university hospital with a diagnosis of COVID-19 between January 1, 2020, and April 30, 2022. The patients’ demographic and clinical characteristics were recorded. The Chi-square, Cox and logistic regression was performed, *P* < 0.05 was considered statistically significant.

**Results:**

Total 1723 patients (50.1% were men, mean age: 60.6 ± 16.90) who had not been vaccinated rate was 27.0% (> 3 doses: 45.7%). Mortality rate was 17.0%. Increasing age, male, a high Charlson Comorbidity Index (CCI), and no vaccination significantly increased mortality (*P* < 0.05). The mortality rate was significantly lower in the chloroquine treatment group than in the other treatment groups. Increasing age, male, and a high CCI were determined to be factors that significantly increased the length of hospital stay (LOHS). LOHS found to be significantly lower in the favipiravir or chloroquine groups compared to the remaining treatment groups (*P* < 0.001). Both mortality and the LOHS significantly differed according to AST, d-dimer, ferritin, and GFR.

**Conclusion:**

This study primarily investigated the effect of treatment and vaccination on the prognosis of COVID-19. This was determined to be prepared for another potential pandemic that may arise due to COVID-19.

## Introduction

The severe acute respiratory syndrome coronavirus-2 (SARS-CoV2) virus caused the development of coronavirus disease 2019 (COVID-19) and resulted in a pandemic by spreading across the world in a short time (Lu et al. 2020). The World Health Organization (WHO) declared COVID-19 as an emergency of international concern (Sohrabi et al. 2020). COVID-19 has affected more than 200 countries, causing the deaths of 6.5 million people in the world, including more than 100 thousand people in Turkey (Chakraborty and Maity 2020; Cascella et al. 2023; Adalja et al. 2020; Zeren and Yilanci 2020). Although all age groups are at risk for contracting COVID-19, it has been observed that especially elderly patients and those with comorbidities such as diabetes, asthma, chronic obstructive pulmonary disorder, hypertension, and coronary artery disease have a more severe disease course (Hernández-Galdamez et al. 2020).

COVID-19 has different clinical manifestations in humans, ranging from mild symptoms, such as weakness and signs of upper respiratory tract infection to mortality due to acute respiratory failure (Wiersinga et al. 2020). With the rapid spread of the disease, agents to be used in the treatment were rapidly tested in clinical studies and introduced into clinical use. Currently, the primary treatments for the disease are antiviral drugs, immunomodulators, neutralizing antibodies, and cell and gene therapies (Niknam et al. 2022). Combination therapies have also been shown to have more advantages (Yuan et al. 2023).

Studies have been conducted to predict the prognosis of COVID-19, and significant results have been obtained regarding lymphopenia and eosinopenia, neutrophil-to-lymphocyte ratio, platelet-to-lymphocyte ratio, ferritin, and d-dimer (Kermali et al. 2020; Lagunas-Rangel 2020; Yao et al. 2020; Valverde-Monge et al. 2021). Male gender, older age, smoking, diabetes, hypertension, and cardiovascular or respiratory system diseases have been determined to be prognostic factors that may cause COVID-19 to have a more severe course and increase the mortality rate (Zhang et al. 2022). In addition, it has been reported that socioeconomic status, diet, lifestyle, geographical differences, ethnicity, viral load exposure, onset of treatment, and quality of healthcare services also affect individual outcomes (Gao et al. 2021). However, only a few studies have investigated the relationship between drugs used in the treatment of COVID-19 and disease prognosis among inpatients (Alotaibi et al. 2021). In addition, considering the increasing vaccination rates across the world, there is a need for a multifactorial study to evaluate the relationship between vaccination and COVID-19 prognosis.

This study aimed to investigate the effects of drugs used in the treatment of COVID-19 (chloroquine and favipiravir), vaccination status and doses, laboratory parameters, demographic characteristics, and the Charlson Comorbidity Index on prognosis [length of hospital stay (LOHS) and mortality] in patients admitted to the hospital with a diagnosis of COVID-19.

## Methods

In this single-center, descriptive, cross-sectional, non-interventional study, the data of inpatients diagnosed with COVID-19 at the Health Application and Research Center of Afyonkarahisar Health Sciences University (AFSU) between January 1, 2020, and April 30, 2022 were retrospectively recorded from the hospital’s electronic system. The study was initiated after the approval of Afyonkarahisar University of Health Sciences (AFSU) Non-Interventional Research Ethics Committee and Ministry of Health (Turkey). In accordance with the Regulation on the Processing and Privacy of Personal Health Data and the principles of the Declaration of Helsinki, all identity information of the patients was anonymized.

The data of a total of 1892 inpatients with COVID-19 were retrospectively collected. Since a patient may have had more than one presentation, each visit was evaluated separately. No sample was selected, and all inpatients treated within 1 year were included in the study.

**Fig. 1:**
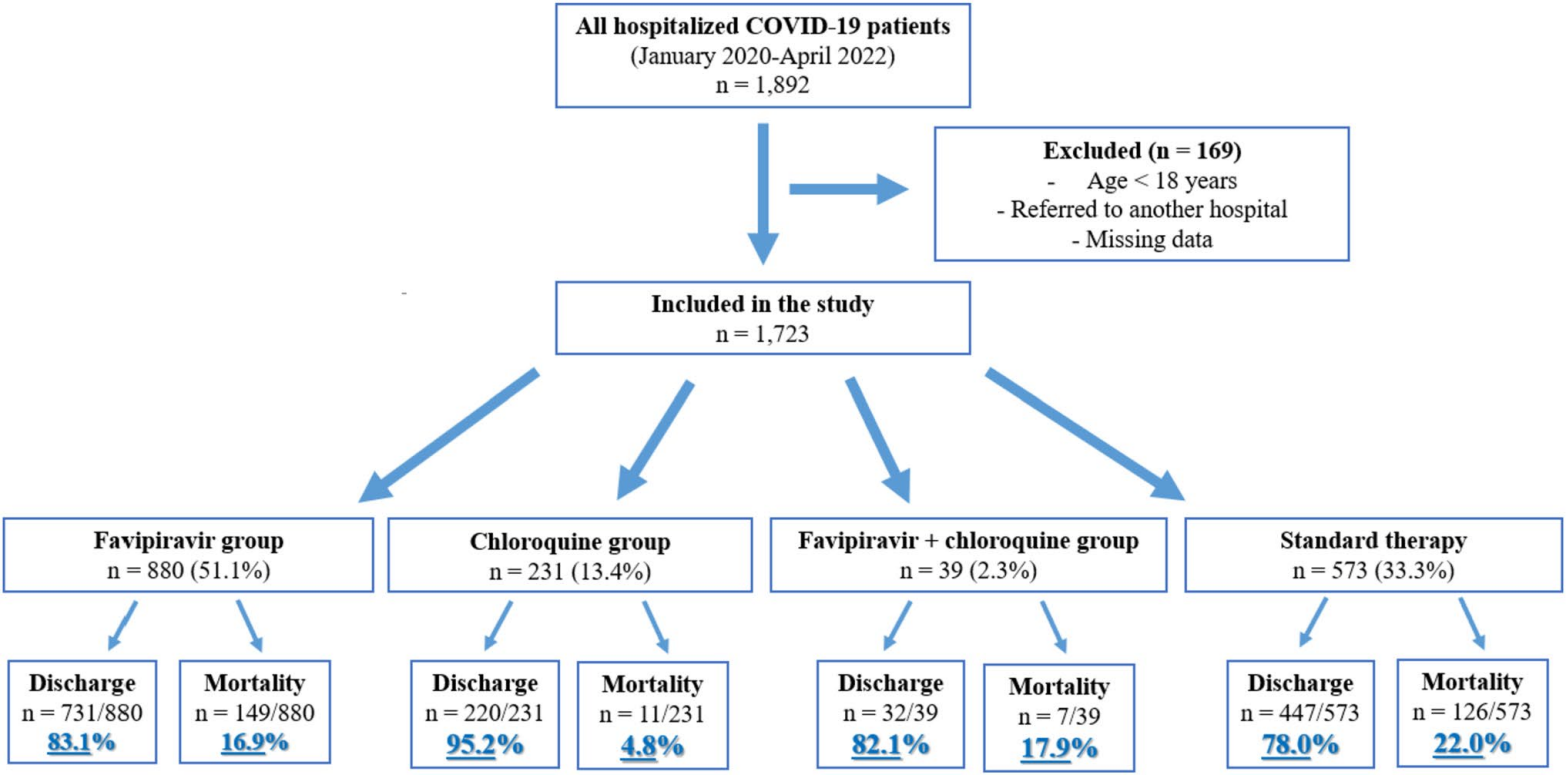
Flow chart of the study population

The inclusion criteria were as follows: (a) diagnosis of COVID-19 based on a positive polymerase chain reaction test or clinical and radiological (thoracic computed tomography or chest X-ray) findings; (b) receiving inpatient treatment; (c) age of 18 years or over; and (d) taking at least one drug during hospitalization. Excluded from the study were patients who were referred to another center and therefore could not be followed up, as well as those with missing data. After applying these criteria, the data of 1723 patients were included in the study (Fig. 1).

The independent variables were the patients’ demographic characteristics (age and gender), comorbidities, Charlson Comorbidity Index (CCI) values, biochemical laboratory findings measured on the first day [creatinine, glomerular filtration rate (GFR), aspartate aminotransferase (AST), alanine transaminase (ALT), d-dimer, and ferritin], COVID-19 vaccine status, and drug therapy used for the treatment of COVID-19. The reference ranges of the hospitals were taken into account when evaluating the laboratory results. The dependent variable was disease prognosis, for which mortality status and LOHS were recorded.

The vaccines administered for COVID-19 in the study group were mRNA-based BioNTech (Pfizer/BioNTech Fosun Pharma), inactivated CoronaVac (Sinovac Research and Development Co. Ltd.), and inactivated Turkovac (Erciyes University and Health Institutes of Turkey). Only the presence/absence of vaccination and the number of vaccination doses (one to four) were recorded for each patient.

Descriptive data were presented as number (*n*), percentage (%), and mean (standard deviation) values. Age, LOHS, and laboratory findings were evaluated by dividing the median values into two groups. CCI, a parameter used to predict 1-year mortality, was analyzed in two groups (CCI < 1 and CCI ≥ 1). COVID-19 treatment was divided into four groups: favipiravir (FVP), chloroquine (CLQ), FVP + CLQ, and standard therapy (ST) (not including FVP or CLQ). The conformity of the data to the normal distribution was checked with the Shapiro–Wilk test. The relationship between dependent and independent variables was evaluated with the Chi-square test. Cox regression analysis was used for mortality, and logistic regression analysis was performed for LOHS. Variables that had a significant relationship in the univariate analysis were included in the multivariate models. For the highly correlated variables, one was included in these models (e.g., GFR or creatinine and AST or ALT). Data were analyzed using SPSS v. 24 (SPSS Inc., Chicago, IL, USA) statistical software. A *P* value of *P* < 0.05 was considered statistically significant.

## Results

### Demographic characteristics of patients

The mean age of the 1723 patients included in the study was 60.6 ± 16.90 years, and 50.1% of the patients (*n* = 863) were male. The most common comorbidity was hypertension at a rate of 23.7%, followed by diabetes mellitus at 15.8%. CCI was calculated to be 0 in 68.8% (*n* = 186) of the patients. The rate of patients who had not been vaccinated for COVID-19 was 27.0% (*n* = 465), while 3.9% (*n* = 68) of the patients had received one vaccination dose, 23.4% (*n* = 403) two doses, and 45.7% (*n* = 787) three or more doses. The rate of patients who received FVP and/or CLQ treatment was 66.7% (*n* = 1150). The median LOHS was 6 days (min: 1-max: 58). Mortality developed in 17.0% (*n* = 293) of the patients.

### Comparison of clinical outcomes

When the factors affecting mortality were examined, it was found that increasing age, male gender, the absence of COVID-19 vaccination, and a high CCI significantly increased mortality, while the mortality rate significantly decreased as the number of COVID-19 vaccination doses increased (linear trend, *P* < 0.001).

There was a significant difference in mortality between the treatment groups (Table 1). When the groups were compared, the mortality rate was significantly lower in the CLQ group than in the remaining treatment groups. There was a significant decrease in mortality in the group that received FVP treatment compared to the group that received ST alone. No significant difference was found in mortality between the remaining treatment groups (not included in tables).

**Table 1:** Comparison of prognosis (mortality/hospital stay) according to independent variables

	Discharge (*n* = 1430)	Mortality (*n* = 293)	*P* value	Hospital stay		*P* value
				≤ 6 days	> 6 days	
Age, *n* (%)						
≤ 63	821 (93.4)	58 (6.6)	< 0.001^*^	520 (59.2)	359 (40.8)	< 0.001^*^
> 63	609 (72.2)	235 (27.8)		321 (38.0)	523 (62.0)	
Gender, *n* (%)						
Female	765 (88.6)	98 (11.4)	< 0.001^*^	472 (54.7)	391 (45.3)	< 0.001^*^
Male	665 (77.3)	195 (22.7)		369 (42.9)	491 (57.1)	
Vaccination status, *n* (%)						
No vaccine	197 (42.4)	268 (57.6)	< 0.001^a, *^	165 (35.5)	300 (64.5)	< 0.001^*^
1 dose	61 (89.7)	7 (10.3)		33 (48.5)	35 (51.5)	
2 doses	390 (96.8)	13 (3.2)		226 (56.1)	177 (43.9)	
≥ 3 doses	782 (99.4)	5 (0.6)		417 (53.0)	370 (47.0)	
CCI, *n* (%)						
< 1	1031 (86.9)	155 (13.1)	< 0.001^*^	631 (53.2)	555 (46.8)	< 0.001^*^
≥ 1	399 (74.3)	138 (25.7)		210 (39.1)	327 (60.9)	
Medication used, *n* (%)						
FVP	731 (83.1)	149 (16.9)	< 0.001^*^	452 (51.4)	428 (48.6)	< 0.001^*^
CLQ	220 (95.2)	11 (4.8)		184 (79.7)	47 (20.3)	
FVP + CLQ	32 (82.1)	7 (17.9)		9 (23.1)	30 (76.9)	
ST	447 (78.0)	126 (22.0)		196 (34.2)	377 (65.8)	

Chi-square analysis was performed

^*a*^Linear by linear association

^*^Statistically significant

*CCI* Charlson Comorbidity Index, *FVP* favipiravir, *CLQ* chloroquine, *ST* standard therapy that did not include FVP or CLQ

The factors affecting LOHS were found to be similar to those affecting mortality. Increasing age, male gender, and a high CCI significantly increased LOHS. When the vaccination rates were examined, LOHS was significantly higher in the unvaccinated group than in all vaccinated groups (Table 1). However, there was no significant difference in LOHS according to the number of vaccine doses received (Chi-square test, *P* > 0.05). The hospital stay was determined to be the shortest in the group that received CLQ treatment. LOHS was lower in the FVP or CLQ treatment groups compared to the remaining treatment groups (*P* < 0.001). Although no significant difference was found between the FVP + CLQ group and the ST group in terms of LOHS, the patients who received FVP + CLQ group had a longer hospital stay compared to those in other treatment groups (Table 1).

Concerning laboratory findings, significant differences were found in both mortality and LOHS in the presence of high AST, d-dimer, and ferritin levels and a low GFR value. Mortality was also significantly affected by a high creatinine level, and LOHS was significantly affected by a high ALT level (Table 2).

**Table 2:** Comparison of laboratory parameters according to dependent variables

	Discharge *n* (%)	Mortality *n* (%)	*P* value	Hospital stay		*P* value
				≤ 6 days	> 6 days	
Creatinine (*n* = 1573)						
≤ 0.74 ng/mL	683 (86.9)	103 (13.1)	< 0.001	371 (47.2)	415 (52.8)	0.216
> 0.74 ng/mL	618 (78.5)	169 (21.5)		396 (50.3)	391 (49.7)	
GFR (*n* = 1547)						
≤ 89	583 (74.5)	200 (25.5)	< 0.001^*^	338 (43.2)	445 (56.8)	< 0.001^*^
> 89	698 (91.4)	66 (8.6)		413 (54.1)	351 (45.9)	
AST (*n* = 1551)						
≤ 23 IU/L	727 (89.4)	86 (10.6)	< 0.001^*^	421 (51.8)	392 (48.2)	0.025^*^
> 23 IU/L	563 (76.3)	175 (23.7)		340 (46.1)	398 (53.9)	
ALT (*n* = 1560)						
≤ 19 IU/L	671 (83.4)	134 (16.6)	0.611	417 (51.8)	388 (48.2)	0.024^*^
> 19 IU/L	622 (82.4)	133 (17.6)		348 (46.1)	407 (53.9)	
D-dimer (*n* = 1412)						
≤ 0.42 μg/mL	665 (93.9)	43 (6.1)	< 0.001^*^	399 (56.4)	309 (43.6)	< 0.001^*^
> 0.42 μg/mL	500 (71.0)	204 (29.0)		289 (41.1)	415 (58.9)	
Ferritin (*n* = 1465)						
≤ 287.7 ng/mL	679 (93.5)	47 (6.5)	< 0.001^*^	445 (61.3)	281 (38.7)	< 0.001^*^
> 287.7 ng/m L	532 (72.0)	207 (28.0)		279 (37.8)	460 (62.2)	

Chi-square analysis was performed

^*^Statistically significant

*GFR* glomerular filtration rate, *AST* aspartate aminotransferase, *ALT* alanine aminotransferase

### Multiple regression analysis for mortality and hospital stay

In the Cox regression analysis, mortality significantly increased with increasing age, AST, and d-dimer values and significantly decreased in the presence of two or more doses of vaccination (Table 3). The logistic regression analysis revealed that increasing age, male gender, a high AST level, and a high ferritin level significantly increased LOHS, while the presence of two or more doses of vaccination or FVP or CLQ treatment significantly reduced LOHS (Table 4).

**Table 3:** Cox regression analysis of mortality

Parameters	*P* value	Odds ratio (95% confidence interval)
Age		
≤ 63 years		1.00
> 63 years	< 0.001^*^	1.81 (1.37–2.40)
Gender		
Female		1.00
Male	0.066	1.26 (0.99–1.61)
Vaccination status		
No vaccine		1.00
1 dose	0.485	0.79 (0.41–1.53)
2 doses	0.010^*^	0.64 (0.45–0.90)
≥ 3 doses	< 0.001^*^	0.54 (0.40–0.72)
CCI		
Index < 1		1.00
Index ≥ 1	0.094	1.25 (0.96–1.63)
Medication		
Standard therapy		1.00
FVP	0.067	0.48 (0.22–1.05)
CLQ	0.871	0.94 (0.43–2.03)
FVP + CLQ	0.749	1.19 (0.41–3.47)
GFR		
≤ 89		1.00
> 89	0.282	1.16 (0.88–1.54)
AST		
≤ 23 IU/L		1.00
> 23 IU/L	0.004^*^	1.53 (1.14–2.04)
D-dimer		
≤ 0.42 μg/mL		1.00
> 0.42 μg/mL	0.010^*^	1.61 (1.12–2.30)
Ferritin		
≤ 287.7 ng/mL		1.00
> 287.7 ng/mL	0.745	1.06 (0.74–1.53)

^*^Statistically significant

*FVP* favipiravir, *CLQ* chloroquine, *CCI* Charlson Comorbidity Index, *GFR* glomerular filtration rate, *AST* aspartate aminotransferase

**Table 4:** Logistic regression analysis of hospital stay

Parameters	*P* value	Hazard ratio (95% confidence interval)
Age		
≤ 63 years		1.00
> 63 years	< 0.001^*^	1.80 (1.35–2.41)
Gender		
Female		1.00
Male	0.033^*^	1.32 (1.02–1.71)
Vaccination status		
No vaccine		1.00
1 dose	0.551	0.81 (0.41–1.61)
2 doses	0.006^*^	0.60 (0.42–0.87)
≥ 3 doses	< 0.001^*^	0.52 (0.38–0.70)
CCI		
Index < 1		1.00
Index ≥ 1	0.056	1.30 (0.99–1.71)
Medication		
Standard therapy		1.00
FVP	< 0.001^*^	0.56 (0.42–0.74)
CLQ	< 0.001^*^	0.13 (0.08–0.20)
FVP + CLQ	0.110	1.95 (0.86–4.43)
GFR		
≤ 89		1.00
> 89	0.176	0.82 (0.62–1.09)
AST		
≤ 23 IU/L		1.00
> 23 IU/L	0.005^*^	1.52 (1.14–2.03)
D-dimer		
≤ 0.42 μg/mL		1.00
> 0.42 μg/mL	0.332	1.13 (0.88–1.46)
Ferritin		
≤ 287.7 ng/mL		1.00
> 287.7 ng/mL	< 0.001^*^	1.84 (1.40–2.41)

^*^Statistically significant

*FVP* favipiravir, *CLQ* chloroquine, *CCI* Charlson Comorbidity Index, *GFR* glomerular filtration rate, *AST* aspartate aminotransferase

## Discussion

In this study, the data of patients who were diagnosed with COVID-19 and received inpatient treatment at a tertiary university hospital were retrospectively obtained. The relationship of treatments and vaccination status with prognosis was examined, and other factors that could affect the prognosis were also included in the study. Although the mean age of the sample was high since patients aged 18 and under were not included in the study, it was similar to patient ages reported in previous studies conducted in Turkey (Gedik et al. 2023; Kokturk et al. 2021). In our study, as in similar retrospective studies, hypertension and diabetes mellitus were determined to be the two most common comorbidities (Fang et al. 2020; Guisado-Vasco et al. 2020). We found the median LOHS to be 6 days, consistent with previous research indicating that this value ranges from 5 to 17 days (Rees et al. 2020; Alwafi et al. 2021; Birhanu et al. 2022; Guo et al. 2021).

Although both drugs are used in the treatment of COVID-19 with their efficacy having been most clearly shown by clinical studies, CLQ treatment has been abandoned over time as studies on the treatment of COVID-19 have increased (Rattanaumpawan et al. 2022; Tawfik et al. 2022; Cai et al. 2020; Udwadia et al. 2021; Chen et al. 2020; Geleris et al. 2020; Ho et al. 2021). Therefore, in the current study, the rate of patients who had received CLQ treatment was found to be much lower.

Mortality rates were reported to be 2% and 4.5% in two studies conducted with inpatients with COVID-19 in Turkey (Kokturk et al. 2021; Fang et al. 2020), and these values were much lower than the rate we found in our study. However, when COVID-19 inpatients from other countries are examined, this rate ranges from 20.2 to 25.7% (Guisado-Vasco et al. 2020; Quah et al. 2020; Horwitz et al. 2021). In a meta-analysis reviewing similar studies on inpatients with COVID-19, an average mortality rate of 17.1% was found (Macedo et al. 2021), which is very similar to the mortality rate obtained from our study.

We found older age to be an independent risk factor for mortality in COVID-19 patients. Although male gender was significant in the univariate analysis of mortality, it lost its significance in the multivariate analysis. In studies evaluating inpatients with COVID-19 in Turkey, increasing age is observed to be one of the significant risk factors for mortality (Kokturk et al. 2021; Medetalibeyoglu et al. 2020; Birtay et al. 2021). Research results from different countries are similar. In a retrospective study of patients hospitalized with COVID-19 in Brazil, Souza et al. determined that older age and male gender were independent risk factors for mortality (Souza et al. 2021). Multicenter retrospective studies of hospitalized patients from Italy and China also suggest that age is a risk factor for mortality (Bellan et al. 2020; Zhou et al. 2020). Similarly, in a systematic review covering 14 clinical trial meta-analyses, it was reported that older age and male gender were factors that significantly increased mortality (Tian et al. 2020). In another systematic review, age was found to be the factor that most affected mortality (Mehraeen et al. 2020). Older age is one of the most important factors affecting prognosis not only in COVID-19 but in many other diseases.

In this study, there was a significant decrease in mortality with an increase in the number of COVID-19 vaccine doses received, and it was observed that having received two or more doses of vaccination was one of the independent factors affecting mortality. In a systematic review comparing the results of clinical studies using different vaccines, it was stated that there was a significant decrease in mortality rates with vaccination (Huang and Kuan 2022). In a large-scale cohort study conducted in Argentina, it was found that three different vaccines developed for COVID-19 disease significantly reduced mortality (Macchia et al. 2021). Although the design and vaccine types of that study were different from those of our research, the authors reached similar results. In a multicenter observational study conducted in the USA, vaccination was determined to reduce mortality among hospitalized patients (Stepanova et al. 2022). In the current study, 70% of the patients had received at least two vaccine doses, and our results emphasized the importance of not only being vaccinated but also receiving a minimum of two vaccine doses in terms of mortality.

CLQ provided a significant decrease in the mortality rate compared to the remaining treatment groups, while FVP resulted in a significant difference only compared to ST, but both CLQ and FVP had any significant effect on mortality according to the multivariate analysis. In a retrospective study evaluating FVP and CLQ treatments, the mortality rates of the FVP and CLQ groups were determined to be 8.2% and 7.3%, respectively, and although there was a significant difference between these two groups, this significance was not observed in the multivariate analysis (Alotaibi et al. 2021), consistent with our findings. In another retrospective study, Alamer et al. reported that there was no significant difference in mortality between the groups receiving FVP and ST (Alamer et al. 2021). In a study comparing the results of CLQ and a placebo on mortality in hospitalized patients, there was no significant difference in mortality (Self et al. 2020). In our study, similar to the literature, two drug treatments, alone or in combination, had no significant effect on mortality. Studies investigating the effects of drugs and their combinations, such as CLQ + azithromycin, lopinavir, ritonavir, remdesivir, and ivermectin, on the prognosis of COVID-19 are ongoing (Bhimraj et al. 2020).

We found age and male gender to be independent risk factors for LOHS. Similarly, in an observational study conducted in the USA, LOHS was found to be significantly higher in male patients (Nguyen et al. 2021). In a retrospective study conducted in Italy, LOHS was significantly higher in women (Fortunato et al. 2021). In a retrospective study from China, the authors determined LOHS to be significantly longer in older individuals but did not find a significant difference according to gender (Wang et al. 2022). The discrepancies in the results reported from different countries can be attributed to the differences in the ethnic origins of patients.

Similar to mortality, LOHS was significantly lower in the patient group that had received two or more doses of vaccination. The hospital stay was shortened by 40% in the presence of two vaccine doses and by almost 50% in the presence of three doses. In a study conducted in Norway, in which the results of patients who had received at least two doses of an mRNA vaccine and those without a COVID-19 vaccination history were compared, a shorter hospital stay and a lower risk of intensive care unit (ICU) admission were reported in the former (Whittaker et al. 2022). A large-scale descriptive study conducted in the USA reported the LOHS of unvaccinated patients to be longer (Havers et al. 2022). In another large-scale study that was conducted in Italy and included different vaccines, unvaccinated, partially vaccinated, and fully vaccinated groups were compared, and while no significant difference was found between the unvaccinated and partially vaccinated groups in terms of LOHS, the hospital stay was found to be significantly shorter in the fully vaccinated group compared to the remaining groups (Cocchio et al. 2022). The results of our study are in agreement with the literature.

Having received CLQ or FVP treatment was one of the independent factors that resulted in a reduction in LOHS. The reduction in LOHS was much more pronounced in the presence of CLQ treatment. In a multicenter randomized clinical trial with a small number of patients, FVP and CLQ were compared, and no significant difference was found in terms of LOHS (Dabbous et al. 2021). Another multicenter retrospective cohort study compared COVID-19 patients who had and had not received FVP therapy in terms of hospital and ICU stays, a significant decrease was found in the FVP group (Mutair et al. 2022). In a multicenter randomized clinical trial comparing FVP and lopinavir + ritonavir, no significant difference was reported in terms of LOHS (Solaymani-Dodaran et al. 2021). In a randomized clinical trial conducted in China, CLQ and lopinavir + ritonavir were compared, and CLQ was found to reduce LOHS (Huang et al. 2020). According to our results, CLQ treatment caused a significant decrease in LOHS.

When the laboratory findings were examined, d-dimer was found to be a significant independent risk factor for mortality, ferritin for LOHS, and AST for both mortality and LOHS. There are many studies and meta-analyses in the literature that support our results. In two different meta-analyses, increased aminotransferase levels were shown to be a risk factor for mortality (Boregowda et al. 2020; Wang et al. 2021). In another meta-analysis, the authors found a significant difference in mortality according to AST, ALT, and D-dimer levels (Loomba et al. 2022). However, there are also studies showing that AST rises more than ALT in COVID-19 patients (Bloom et al. 2021). In studies conducted with hospitalized patients in different countries, ferritin has been shown to be an indicator for predicting in-hospital mortality (Tural Onur et al. 2021; Lino et al. 2021; Deng et al. 2021; Ahmed et al. 2021). In contrast, in our study, ferritin did not significantly predict mortality as an independent factor, despite being an independent risk factor for LOHS. We determined d-dimer to be an independent risk factor for mortality, as supported by many previous studies in the literature (Sakka et al. 2020; Zhang et al. 2020; Shah et al. 2020).

In this study, a comparison between different vaccines was not made, and confounding factors, such as the onset of COVID-19 treatment in hospitalized patients, drug doses, and comorbidities, were not included in statistical analyses. In addition, the study was conducted with hospitalized patients without making any distinction according to ward or ICU admission, and no COVID-19 disease severity classification was undertaken. The most important reason for these limitations was the retrospective design, which resulted in an inability to access all related data.

## Conclusion

COVID-19, a disease with a high risk of transmission that can be fatal, especially in elderly patients, has caused the deaths of millions of people across the world. Although current vaccines prevent the development of the virus, their effects when combined with treatment remain a matter of debate. In this study, the effects of treatment and vaccination on the prognosis of COVID-19 were primarily investigated. In addition to using FVP and CLQ alone and together, investigating the impact of COVID-19 vaccine dose on prognosis along with treatment and finding significant outcomes represents the rich aspect of this study and provides new information for future research. Furthermore, the association between the most common demographic and laboratory characteristics and prognosis has been investigated in the literature, and the results we found in our study support previous studies. To be prepared for another potential pandemic that may arise due to COVID-19, there is a need for further research both in Turkey and in other countries to evaluate the effects of vaccination and treatment on disease prognosis.

## Data Availability

Data are available from the authors upon reasonable request.
